# The Relationship between Intrinsic Couplings of the Visual Word Form Area with Spoken Language Network and Reading Ability in Children and Adults

**DOI:** 10.3389/fnhum.2017.00327

**Published:** 2017-06-23

**Authors:** Yu Li, Linjun Zhang, Zhichao Xia, Jie Yang, Hua Shu, Ping Li

**Affiliations:** ^1^National Key Laboratory of Cognitive Neuroscience and Learning and IDG/McGovern Institute for Brain Research, Beijing Normal UniversityBeijing, China; ^2^Department of Cognitive Science and ARC Centre of Excellence in Cognition and its Disorders, Macquarie UniversitySydney, NSW, Australia; ^3^Faculty of Linguistic Sciences and KIT-BLCU MEG Laboratory for Brain Science, Beijing Language and Culture UniversityBeijing, China; ^4^Department of Psychology and Center for Brain, Behavior and Cognition, Pennsylvania State University University Park, PA, United States

**Keywords:** reading ability, language network, visual word form area, Chinese reading, left posterior middle frontal gyrus

## Abstract

Reading plays a key role in education and communication in modern society. Learning to read establishes the connections between the visual word form area (VWFA) and language areas responsible for speech processing. Using resting-state functional connectivity (RSFC) and Granger Causality Analysis (GCA) methods, the current developmental study aimed to identify the difference in the relationship between the connections of VWFA-language areas and reading performance in both adults and children. The results showed that: (1) the spontaneous connectivity between VWFA and the spoken language areas, i.e., the left inferior frontal gyrus/supramarginal gyrus (LIFG/LSMG), was stronger in adults compared with children; (2) the spontaneous functional patterns of connectivity between VWFA and language network were negatively correlated with reading ability in adults but not in children; (3) the causal influence from LIFG to VWFA was negatively correlated with reading ability only in adults but not in children; (4) the RSFCs between left posterior middle frontal gyrus (LpMFG) and VWFA/LIFG were positively correlated with reading ability in both adults and children; and (5) the causal influence from LIFG to LSMG was positively correlated with reading ability in both groups. These findings provide insights into the relationship between VWFA and the language network for reading, and the role of the unique features of Chinese in the neural circuits of reading.

## Introduction

Reading is a skill unique to humans and it plays a critical role in education and communication in modern society. It is universally accepted that learning to read reorganizes the existing brain structures that have other functions. In other words, learning to read is a process of brain reorganization (Dehaene and Cohen, 2007). The visual word form area (VWFA) located in the left ventral occipito-temporal cortex is widely thought to be associated with literacy acquisition across different writing systems (Cohen et al., 2000; Monzalvo and Dehaene-Lambertz, 2013; Dehaene et al., 2015; Dehaene and Dehaene-Lambertz, 2016; Saygin et al., 2016). Increasing evidence from studies of dyslexia and normal reading development has also pointed to the core function of VWFA in skilled reading (Dehaene and Cohen, 2011; Price and Devlin, 2011; Peterson and Pennington, 2012).

As reading is a process that maps written symbols to spoken language, learning to read is a process of establishing solid and stable interactions between written and spoken languages through training and learning experiences. In the brain, reading acquisition changes the functions and structures in the language-related brain areas (Carreiras et al., 2009; Dehaene et al., 2010; Monzalvo and Dehaene-Lambertz, 2013), and strengthens the connections between VWFA and spoken language regions including Broca’s and Wernicke’s areas responsible for articulation and auditory processing (e.g., Thiebaut de Schotten et al., 2014). For example, an fMRI study has shown that children who received reading instruction have higher regional activation in the spoken language network than children who didn’t receive reading instruction (Monzalvo and Dehaene-Lambertz, 2013). Compared with illiterates, literates/ex-illiterates who learn to read during childhood/adulthood show an increase in fractional anisotropy in the temporo-parietal portion of the left arcuate fasciculus which connects VWFA and a temporal language area (Thiebaut de Schotten et al., 2014). The findings in skilled readers using functional/structural connectivity and lateralization analysis methods also consistently indicate strong connectivity between VWFA and the spoken language network (Cai et al., 2008; Koyama et al., 2011; Wandell et al., 2012; Wang et al., 2012; Bouhali et al., 2014; Zhang et al., 2014; Abboud et al., 2015). For examples, Abboud et al. (2015) found that VWFA shows more intrinsic functional connections with the left temporal cortex and the left inferior frontal gyrus (LIFG). Bouhali et al. (2014) revealed that VWFA showed dense anatomical connections with anterior language areas; Cai et al. (2008) found that the posterior occipito-temporal areas involved in visual word processing are lateralized to the same hemisphere involved in language production, indicating that these two systems are strongly attached in the reading brain.

Proficient reading has to be achieved through a significant amount of training in schools or other learning experiences. On the one hand, long-term reading training inevitably changes the connections between VWFA and spoken language areas (Thiebaut de Schotten et al., 2014). On the other hand, the strengthened connections are highly linked to reading ability (Koyama et al., 2011; Wang et al., 2012; Zhang et al., 2014) while aberrant connections may indicate reading disability (van der Mark et al., 2011;Finn et al., 2014;Schurz et al., 2015). For instance, Koyama et al. (2011) found that the strengths in resting-state functional connectivity (RSFC) between VWFA and IFG and the inferior parietal lobule are highly correlated with reading ability in both child and adult readers. Finn et al. (2014) found that the functional connectivity patterns among key reading areas are disrupted or reduced in dyslexics. Taken together, a clear picture based on the existing literature is that the connections between VWFA and spoken language areas in the frontal and temporal regions are reinforced by reading experience and these connections index reading ability.

However, it remains unclear what developmental differences in these resting-state connections exist between adults and children and what relationships are there between RSFC and reading competence. A number of questions are currently associated with the limited literature. In a RSFC study of English reading, Koyama et al. (2011) found that the RSFC between VWFA and spoken language areas was highly correlated with reading ability in both children and adult group, but the patterns were very different between these two groups. The correlation was negative in children while it was positive in adults, suggesting that the VWFA and the language network RSFC and its relationship with reading ability are not stable, and possibly change with age. Vogel et al. (2012) found that compared with children, young adults showed stronger RSFC between VWFA and spoken language areas and children didn’t show significant connectivity between them. Finn et al. (2014) found that dyslexic groups of different ages showed very different patterns of abnormality compared with age-matched controls, indicating clear developmental changes. Taking these discrepancies as a starting point, the current study sought to examine the difference in the VWFA-language network connectivity patterns and their relationships with reading ability, in both adult and child readers. We used RSFC and Granger Causality Analysis (GCA) approaches in our study.

RSFC focuses on low-frequency BOLD signal fluctuations (≈0.01–0.1 Hz) of the brain during rest. The study of RSFC patterns for language and cognition has become popular in recent years, and RSFC has been shown to be highly correlated with cognitive variables and behavioral performance (Friston, 2005, 2011; Hampson et al., 2006; Koyama et al., 2011). However, RSFC analysis is based on unidirectional correlations, and therefore cannot reveal directional influences from one brain area to another. To address this issue, researchers in recently years have used Granger causal analysis (GCA) which focuses on directional influences between different areas (Granger, 1969). Recent work using this GCA has generated insights into the functional organization of both healthy and diseased brain networks (Bressler et al., 2008; Hamilton et al., 2011; Liao et al., 2011; Wen et al., 2012, 2013). In our view, these two analytic methods are complementary and could help to clarify the connections and interactions between VWFA and language areas that are associated with reading performance. Specifically, we adopted seed-based RSFC to explore the connections between VWFA and language areas, and the resting-state GCA to investigate the modulatory effect of higher-level language areas on VWFA.

Given the previous findings as reviewed (Koyama et al., 2011; Vogel et al., 2012; Finn et al., 2014), we hypothesize that the directional and unidirectional patterns of connectivity between VWFA and language areas serve to index reading ability in both child and adult groups, but the two groups may differ to some extent in the specific connectivity patterns. This age-effect is considered particularly in the context of the discussion of the nature of the Chinese logographic writing system, that is, whether reading-related brain connectivity patterns reflect language-specific features of Chinese vs. alphabetic systems (Tan et al., 2005; Perfetti and Tan, 2013; Perfetti et al., 2013). Specifically, there has been increasing evidence that Chinese reading is highly related to handwriting in Chinese (Tan et al., 2005, 2013; Perfetti and Tan, 2013; Cao and Perfetti, 2016) and activation of the left posterior middle frontal gyrus (LpMFG) or Exner’s area (a handwriting area) has been reported in previous studies of Chinese reading (Siok et al., 2004; Nakamura et al., 2012; see also Cao and Perfetti, 2016). If our findings demonstrate language specificity, we expect brain activity in LpMFG or Exner’s area in the current study. Thus, findings from the current study can help to reveal the neural circuits related to Chinese reading and provide insights into how successful reading in adulthood develops from less skilled reading in childhood.

## Materials and Methods

### Participants

Thirty-two elementary school students (1 from grade 4, 24 from grade 5 and 7 from grade 6) and 30 college students (young adults) participated in this study. Some children and adults from this population also participated in our previous studies (see Zhou et al., 2015, 2016). The university students have received more than 12 years of school education while the elementary school students had been learning to read and write Chinese for 4–6 years. All participants had normal reading performance, normal or corrected-to-normal vision, and had no history of neurological or psychiatric disorders; children had normal verbal and performance IQ; adults have normal IQ as they were students from two major universities (i.e., Beijing Normal University and Beijing University of Posts and Telecommunications) and had to get high scores in the entrance exams (see Table 1 for details of the participants). The current study was approved by the Research Ethics Committee of the National Key Laboratory of Cognitive Neuroscience and Learning at Beijing Normal University (BNU). A written informed consent was obtained from the participants or their legal guardians. The MRI data from four adults and six children were excluded from further analyses because of the participants’ excessive head motion; the final analysis included data from 26 children and 26 adults (see Table 1 for details of the participants).

**Table 1:** Demographic information and cognitive measures (range, ±SD) for each age group.

	Children (26)	Adults (26)	*t*-value
Age (years)	11.35 (10.55–12.60, ±0.53)	21.92 (18–28, ±2.46)	21.385***
Gender (M/F)	13 M/13 F	12 M/14 F	N/A
Verbal IQ	105.9 (89–122, ±7.7)	N/A	N/A
Performance IQ	106.5 (84–128, ±9.7)	N/A	N/A
Word list reading	94.60 (63.59–146.30, ±17.80)	142.05 (89.54–196.79, ±22.87)	8.349***

***p < 0.001.

### Reading Ability Assessment

Reading ability in both children and adults was measured by a word list reading test that has been used and validated in our previous research for testing Chinese reading ability (Zhang et al., 2012; Xia et al., 2016). Word list reading test is considered as a good measure of reading ability across different age groups because it focuses on reading speed and involves the processes of rapid orthography-to-phonology mapping and semantic processing. Similar measures have also been used in other important reading test batteries for Western languages (e.g., Torgeson et al., 1999). Our test included 180 two-character words composed of simple and familiar characters. All participants were asked to name the items as quickly as possible, and there was no time limit in this test. Each word, if correctly read, scored one point. The final score was how many words a participant read correctly in 1 min.

### MRI Data Acquisition

MRI data were obtained on a Siemens MAGNETOM Trio 3-T scanner equipped with a standard quadrature head coil at the BNU Imaging Center for Brain Research. For resting-state functional data, 240 echo-planar imaging (EPI) functional volumes were acquired with 33 axial slices. The entire resting-state continuous scan lasted 8 min. The following scan parameters were used: TR = 2000 ms, TE = 30 ms, flip angle = 90°, slice thickness = 4 mm, matrix size = 64 × 64, FOV = 200 mm × 200 mm, voxel size = 3.1 mm × 3.1 mm × 4.0 mm. During the scan, participants were instructed to relax with eyes closed, keep their head still, and not to think about specific ideas or things (but not to fall into sleep either). After the scan, a simple questionnaire was given and none of the participants reported falling asleep during the scanning. For spatial normalization and localization, T1-weighted anatomical images were collected after the functional scans, using a magnetization prepared rapid gradient-echo (MPRAGE) sequence with 128 sagittal slices. The following scan parameters were used: TR = 2530 ms, TE = 3.39 ms, TI = 1100 ms, flip angle = 7°, FOV = 256 mm × 256 mm; voxel size = 1.3 mm × 1.0 mm × 1.3 mm.

### MRI Data Preprocessing

SPM5^1^ and the Data Processing Assistant for Resting-State fMRI (DPARSF; Chao-Gan and Yu-Feng, 2010) were used to pre-process the resting-state functional MRI data in the following steps. The first 10 volumes were discarded to obtain steady BOLD signal and then the image data were corrected for slice timing and head motion. After that, each participant’s EPI data were spatially normalized to the MNI space using T1 image unified segmentation, and the voxel size was resampled to 3 × 3 × 3 mm. A 6-mm FWHM Gaussian kernel was applied to smooth the EPI data. Linear trends were removed and band-pass temporal filtering (0.01–0.1 Hz) was applied. Several nuisance covariates were regressed out to control for the effects of physiological processes and head motion (namely six head-motion parameters, the white matter signal, the cerebrospinal fluid signal and the global signal). To minimize the influence of head motion on RSFC, we adopted the method proposed by Power et al. (2012), according to which image time points with frame-wise displacement >0.5 mm and their neighboring time points (one time point before and two time points after the bad time points) were discarded because they are considered potentially contaminated with motion artifacts. Using this method, no more than 2 percent of the time points were removed in the time series data of each participant.

### Definition of Language Network and VWFA Coordinates

**Figure 1:**
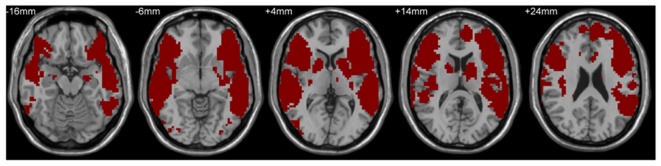
Language network mask based on the combined resting-state functional connectivity (RSFC) maps of left inferior frontal gyrus (LIFG), pars triangularis/pars opercularis and the left posterior superior temporal gyrus in the adult and child groups.

A mask including classical Broca’s and Wernicke’s areas was used to define the language network by the following steps. First, LIFG, pars triangularis/pars opercularis (two subregions), and the left posterior superior temporal gyrus (LpSTG) were chosen from the Harvard-Oxford atlas (see details at http://neuro.imm.dtu.dk/wiki/Harvard-Oxford_Atlas) as Broca’s area and Wernicke’s area, respectively, and the RSFCs of these three areas with the rest of the whole brain were calculated. Subsequently, the false discovery rate correction (FDR, Q value = 0.01, cluster size >100 voxels, two-tailed) was used to identify maps showing statistically significant functional connectivity with the three areas. Finally, the maps corrected by FDR were combined together to get the language network mask (see Figure 1), which was adopted in the following behavior-brain correlation and group comparison analyses. The VWFA location (MNI coordinates, *x* = −52, *y* = −56, *z* = −9) was defined from a meta-analysis of Chinese reading by Bolger et al. (2005) and the coordinates have been based on our previous studies (e.g., Wang et al., 2012, 2015; Zhang et al., 2014).

### RSFC-Behavior Correlation and RSFC Group Comparison Analyses

The Resting-State fMRI Data Analysis Toolkit (REST; Song et al., 2011) and DPARSF (Chao-Gan and Yu-Feng, 2010) were used to calculate RSFC. All brain maps were displayed by the REST Slice Viewer (Song et al., 2011) and BrainNet Viewer (Xia et al., 2013). The mean time-serial signals across voxels within a 6 mm-radius spherical ROI centered on the VWFA coordinates were first calculated. Then the RSFCs of VWFA with the language network for each participant were calculated, producing RSFC r-maps in which the r-value of each voxel represented the extent to which its activity synchronized with the VWFA. The r-maps were then converted into z-maps with Fisher’s r-to-z transformation, which generated approximately normally distributed values that were used for further statistical analyses. Finally, an independent two-sample *t*-test was conducted to find the difference between the adult and child groups in the VWFA-language network RSFC strength; correlations between the interregional RSFC strength and reading performance were computed for each group to find possible connections between language network and VWFA that index reading ability.

### Resting-State Granger Causality Analysis (GCA)

GCA is used for investigating whether the past value of one time-series predict the current value of another time-series. If the current value of time-series *A* can be more accurately estimated by the combination of the past value of time-series *B* and *A* than the past value of only time-series *A*, the time series *A* is thought to be caused by time-series *B* (Granger, 1969). GCA as a data-driven approach has been widely used in neuroimaging studies and demonstrated as an effective method to uncover the interregional causal relationships in the brain (e.g., Goebel et al., 2003; Bressler et al., 2008; Hamilton et al., 2011; Jiao et al., 2011; Wen et al., 2012, 2013). In the current study, coefficient-based GCA with a ROI-wise approach was conducted for each participant using the GCA toolbox implemented in REST (Song et al., 2011; Zang et al., 2012). ROIs were selected based on the results of the RSFC-behavior correlation analysis, including LIFG, left supramariginal gyrus (LSMG) and LpMFG whose RSFCs with VWFA showed significant correlations with reading performance in the adult group or both groups. Each of the spherical ROIs had a 6-mm radius and the mean signal was extracted across all voxels for the following analysis. First, the signed-path coefficients representing bi-directional causal connections at rest between these ROIs were calculated for each group (see Chen et al., 2009; Zang et al., 2012 for method details). GCA-behavior correlation analysis was then performed to look for paired directional connections that correlated with reading performance in each group. If the correlation between reading performance and a causal influence, e.g., from *A* to *B*, is significantly positive, it means that the stronger the causal relation from *A* to *B*, the higher the reading performance. In contrast, if the correlation is significantly negative, it means that the stronger the causal relation from *A* to *B*, the lower the reading performance.

## Results

### Behavioral Results

Mean scores (±SD) of the word list reading test for children and adults were 94.6 (±17.80) and 142.05 (±22.87), respectively (see Table 1). A two-sample *t*-test revealed significant difference between the two groups (*t*_(1,50)_ = 8.349, *p* < 0.0001).

### Group Comparison and RSFC-Behavior Correlations

**Figure 2:**
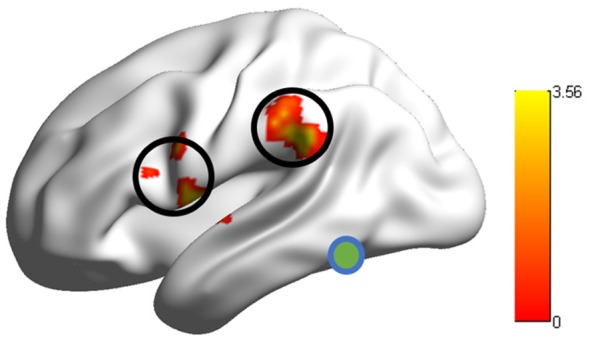
RSFCs between the visual word form area (VWFA) and the LIFG/supramariginal gyrus (LSMG) showed significant differences between adults and children. Colour bar denotes *t*-values.

**Figure 3:**
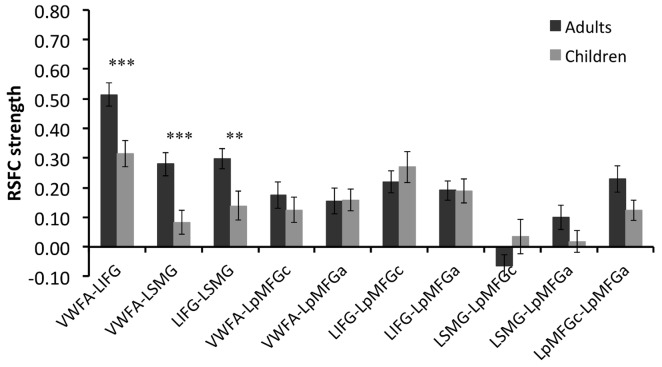
RSFC strength (z score) for each connection in two groups and statistical results of two-sample *t*-tests between two groups. VWFA, visual word form area; LIFG, left inferior frontal gyrus; LSMG, left supramariginal gyrus; LpMFGc, left posterior middle frontal gyrus in child group; LpMFGa, left posterior middle frontal gyrus in adult group. **p < 0.01, ***p < 0.001.

Group comparison in the RSFCs between VWFA and the spoken language network showed that RSFCs of VWFA with LIFG/LSMG were stronger in adults than in children (LIFG, *x, y, z* = −54, 0, 9, *t* value = 3.387, *p* < 0.001, cluster size = 1782 mm^3^; LSMG, *x, y, z* = −57, −36, 27, *t* value = 3.524, *p* < 0.001, cluster size = 1431 mm^3^). LIFG also had stronger connectivity with LSMG in adults than in children (*t* value = 2.71, *p* < 0.01; see Figures 2, 3 and Supplementary Table S1). No other significant differences were found between the groups (*p* > 0.05). Further, one-sample *t*-tests found that the RSFCs between these areas were all significantly different from zero (adults, *t* > 7.06, *p* < 0.05; children, *t* > 2.08, *p* < 0.05), indicating that the connections between VWFA and the spoken language network existed in children, although the strength was weaker than that in adults (see Figure 3 and Supplementary Table S1).

**Figure 4:**
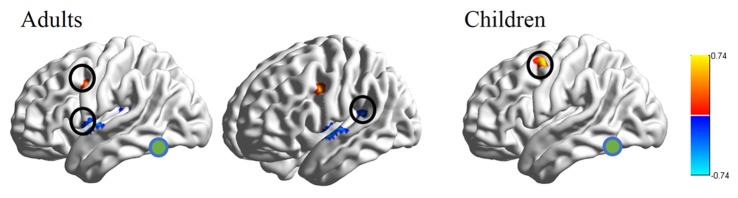
RSFC-behavior correlational maps within language network mask in adults and children, respectively. Green node, VWFA; orange, positive correlations; blue, negative correlations.

**Figure 5:**
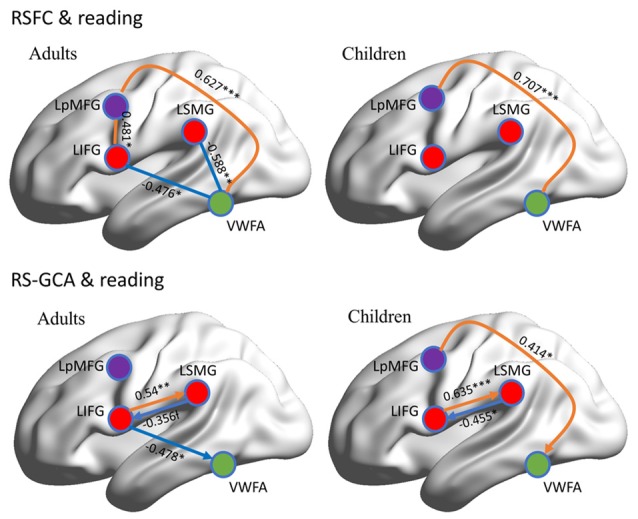
Correlations between RSFC/RS-granger causality analysis (GCA) and reading ability in adult and child groups. Orange color denotes positive correlation; blue color denotes negative correlation. VWFA, visual word form area; LIFG, left inferior frontal gyrus; LSMG, left supramariginal gyrus; LpMFGc, left posterior middle frontal gyrus in child group; LpMFGa, left posterior middle frontal gyrus in adult group. ^†^*p* < 0.1, *p < 0.05, **p < 0.01, ***p < 0.001.

RSFC-behavior analysis of VWFA with the language network was separately performed for the child and adult groups. Results showed that: (1) for adults, the RSFCs of VWFA with LIFG and LSMG negatively correlated with word list reading score (LSMG, *x, y, z* = −33, −36, 12, *r* value = −0.588, *p* < 0.05, cluster size = 918 mm^3^; LIFG, *x, y, z* = −54, 1, 7, *r* value = −0.476, *p* < 0.05, cluster size = 1323 mm^3^), while for children, no significant correlation was found (*p* > 0.05); and (2) for both adults and children, the RSFCs of VWFA with LpMFG were positively correlated with word list reading score (adults, *x, y, z* = −30, 0, 33, *r* value = 0.627, *p* < 0.001, cluster size = 837 mm^3^; children, *x, y, z* = −36, 0, 63, *r* value = 0.707, *p* < 0.001, cluster size = 1539 mm^3^; see Figures 4, 5 and Supplementary Figure S1). One-sample *t-tests* showed that for each group, the RSFC between VWFA and LpMFG was significantly different from zero (adults, *t* = 4.246, *p* < 0.001; children, *t* = 3.913, *p* < 0.001) and the strength was almost equal to each other, indicating that the connection between VWFA and LpMFG existed in both groups (see Figure 3 and Supplementary Table S1). In addition, the LpMFG-LIFG connectivity positively correlated with reading ability in the adult group (*r* value = 0.481, *p* < 0.05) but not in the child group (*p* > 0.05; see Figures 4, 5 and Supplementary Figure S1). Although the peak coordinates of these two LpMFG clusters were not exactly the same in the two groups, they overlapped with or were close to the Exner’s writing area whose peak coordinates are *x* = −22, *y* = −8, *z* = 54 based on a recent meta-analysis (Planton et al., 2013). To further examine this pattern, we obtained two separate clusters of the writing area from nine fMRI or TMS studies involved in writing (see Supplementary Figure S2 and Supplementary Table S2). As can be seen in the Supplementary Figure S2, the child’s LpMFG overlapped with the superior area, whereas the adult’s LpMFG overlapped with the inferior area.

### Resting-State Granger Causal Analysis (GCA)

To better understand the relationship between the causal influences among brain areas and their relationships with reading performance, correlations between causal influence values among the four seeds obtained from RSFC-reading correlation analysis, i.e., VWFA, LIFG, LpMFG, and LSMG and word list reading scores, were calculated (see Figure 5 and Supplementary Figure S3). Results showed that: (1) the LIFG-to-LSMG connectivity was positively correlated with reading ability in both groups (adults, *r* value = 0.54, *p* < 0.001; children, *r* value = 0.635, *p* < 0.001), indicating that the stronger the influence from LIFG to LSMG, the better the reading performance; but the reverse connectivity from LSMG to LIFG was negatively correlated with reading ability (significant for children, *r* value = −0.455, *p* < 0.05, and marginally significant for adults, *r* value = −0.356, *p* = 0.087), indicating that the stronger influence from LSMG to LIFG, the worse the reading performance; (2) the LpMFG-to-VWFA connectivity was positively correlated with reading score in children (*r* value = 0.414, *p* < 0.05) but not in adults (*p* > 0.05), indicating that the stronger the influence from LpMFG to VWFA is associated with enhanced reading performance in developing readers; and (3) the LIFG-to-VWFA connectivity was negatively correlated with reading score only for adults (*r* value = −0.478, *p* < 0.05) but not for children (*p* > 0.05), indicating that the stronger the influence from LIFG to VWFA is associated with worse reading performance. No statistically significant results were found in other connections (*p* > 0.05).

## Discussion

As a complex skill unique to humans, reading recruits a network of brain regions involved in visual analysis and spoken language processing. In particular, reading engages the processes of orthography-to-phonology and phonology-to-semantics mappings that are experience-dependent, and these mappings have been captured by spontaneous brain activity, as previous research has shown (Schlaggar and Church, 2009; Koyama et al., 2011; Vogel et al., 2012; Wang et al., 2012; Schurz et al., 2015). The current study aimed to reveal the neural circuits of skilled reading by investigating the developmental differences between children and adults in the resting-state functional and effective connectivity patterns of the VWFA with spoken language areas (specifically the LIFG and LSMG), and their roles in reading performance. Our findings showed that: (1) the spontaneous connectivity between VWFA and spoken language areas (i.e., LIFG/LSMG) was stronger in adults compared with children; (2) the spontaneous functional patterns of connectivity between VWFA and the language network was negatively correlated with reading ability in adults but not in children; (3) the causal influence from the LIFG to VWFA was negatively correlated with reading ability only for adults but not for children; (4) the RSFCs between LpMFG, VWFA and LIFG were positively correlated with reading ability in both adults and children; and (5) the causal influence from LIFG to LSMG was positively correlated with reading ability in both groups. These patterns of resting-state connectivity contributed to our knowledge of reading and its neural circuits.

### Developmental Changes in VWFA-Language Network Connection

Reading is a skill that needs much experience and extensive training, particularly to establish stable and strong connections between VWFA responsible for visual form processing and the language network responsible for phonological and semantic processing (which includes at least the LIFG and LSMG, as examined in this study). Our results revealed that children have showed significant connections between VWFA and the spoken language areas, in contrast to what was found in Vogel et al. (2012). The inconsistency might be related to the age difference of the children in the two studies with age ranged 10.55–11.6 years in the current study and 6–9 years in Vogel et al. (2012). These results seem to indicate that the functional connectivity between VWFA and spoken language areas is not well-formed in children with only 0–3 years reading experience but emerges after 5–6 years reading training. That is, the construction of the functional connections between these two systems is a gradual and slow process. The connections between the two systems become stronger with more reading experience, which was demonstrated by our finding that there were stronger RSFCs of VWFA-LIFG and VWFA-LSMG in adults compared with children.

The developmental changes in the connections between the reading-specific system (i.e., VWFA) and the spoken language system play a crucial role in reading development. These connections can serve to index reading ability in both normal (Koyama et al., 2011; Vogel et al., 2012) and dyslexic readers (van der Mark et al., 2011; Finn et al., 2014). In the current study, there were negative correlations between the RSFCs of VWFA-LIFG/LSMG and reading ability in adults. The correlation patterns are similar to the findings in English child readers, which revealed that the RSFCs between fusiform gyrus and LIFG/inferior parietal lobule (an area including LSMG) were negatively correlated with reading ability in children (Koyama et al., 2011). Considering the logographic nature of the Chinese writing system, it is possible that the immature RSFC-reading relationship in English reading resembles the mature relationship in Chinese reading.

LpSTG/LSMG is thought to be involved in assembled phonological representations in which grapheme-to-phoneme conversion can be performed in alphabetic reading. But in logographic Chinese reading, it is hard to recode graphic form into phonology because of many homophones and complex inner structures of characters in the Chinese writing system (Tan et al., 2005; Perfetti et al., 2013). Because this area cannot be recruited to retrieve addressed phonological information in Chinese, less suppression of LSMG may impede skilled Chinese reading. Increasing evidence has shown that activation of LSMG was lower during reading-related tasks in adult Chinese readers compared with child readers (e.g., Cao et al., 2010; Brennan et al., 2013). In two recent studies comparing the difference in developmental changes between English and Chinese reading, increased activation of LSMG from children to adults was found in English reading, while decreased activation of LSMG was found in Chinese reading (Cao et al., 2015; Wang et al., 2015). These authors also found similar patterns for LSTG or LpSTG. These results indicate that decreased activation of LSTG/LSMG is associated with skilled Chinese reading. The role of suppressed LSMG activation in Chinese reading can be properly integrated with our finding in adults that the RSFC between VWFA and LSMG was negatively correlated with reading ability. It is likely that the suppressed activation of LSMG is highly tightened up with the suppressed connection of LSMG with VWFA, which jointly facilitates skilled Chinese reading. It is the strong functional connectivity between these two areas that provides the basis of the tight relationship between more suppressed connection of LSMG with VWFA and better reading. In other words, the strong connectivity between them provides a possibility that the suppressed connection of LSMG with VWFA facilitates reading performance in adults. However, a direct comparison between the neural circuits of Chinese and alphabetic reading, especially the connections between VWFA and language network, would be more helpful to reveal the differences in the reading brain.

Furthermore, the negative correlation between the VWFA-LIFG RSFC and reading performance in adults indicates that although LIFG is involved in phonological manipulation and articulation in both English and Chinese, skilled Chinese reading is realized by very different circuits in the brain probably because of the different writing systems of the two languages (see the following “Discussion” Section).

### Developmental Changes of Connections Within the Language Network

Reading is a process of linking elements from written symbols to spoken language. In other words, reading ability depends on language ability to a great extent. Our results revealed that both children and adults showed significant connections between the spoken language areas but the RSFC strength in the adult group is significantly higher than that in the child group, indicating the developmental change from unskilled to skilled reading. This is in line with the finding from a developmental fMRI study of language network (Friederici et al., 2011) that clearly showed stronger connectivity between LIFG and LpSTG/LSMG in skilled adult readers (26 years old) than that in children (5.8 years old) with less reading experience.

Our resting-state GCA results showed that the causal influence from LIFG to LSMG was positively correlated with word-level reading ability, indicating that LIFG plays a key role in modulating the activity of LSMG at rest and this modulation indexes reading ability. These results are consistent with the previous findings that functional and anatomical connections between these two areas are disrupted in dyslexic individuals (e.g., Vandermosten et al., 2012; Boets et al., 2013; Finn et al., 2014). For example, Boets et al. (2013) found that the strength of functional connectivity between LIFG and LSTG is negatively associated with phonemic discrimination reaction time which is a good index of reading ability, suggesting the important role of the connection in skilled reading. According to the dual-stream model of speech processing in the brain outlined by Hickok and Poeppel (2007), LIFG is an area responsible for phonological manipulation and articulation while the posterior parieto-temporal cortex including LSMG is a sensorimotor interface responsible for transferring auditory information to articulatory representations. These two language areas are tightly correlated with each other in the time series of activation during speech perception and production (Buchsbaum et al., 2001). Therefore, the underlying mechanism for the positive relationship between LIFG-to-LSMG causal connectivity and reading ability might be that LIFG takes the responsibility of on-line correction in response to the information from LSMG. But more casual influence from LSMG to LIFG is associated with poorer reading performance, which was demonstrated by our finding that the opposite causal influence from LSMG to LIFG negatively correlated with reading ability in both groups.

Language network constructed mainly by the connections between the left inferior frontal cortex and posterior temporal cortex has already existed although immature in 2-day-old infants (Perani et al., 2011) and in preschool children (Friederici et al., 2011; Xiao et al., 2016). Spoken language acquisition emerges earlier than reading acquisition, and therefore language acquisition provides a prerequisite for reading acquisition. Our results showed that spoken language network functions effectively in reading even when the connections between reading system and spoken language areas are still weak in children. Reading acquisition also reorganizes language network functionally and anatomically (Carreiras et al., 2009; Dehaene et al., 2010; Monzalvo and Dehaene-Lambertz, 2013). The relationships between language and reading acquisition, and their mutual influences in changing brain functions need to be further examined.

### Role of Writing Area in Chinese Reading

An interesting finding of the current study is that the connectivity of VWFA with LpMFG was highly correlated with reading ability in both adults and children, and furthermore, the causal influence from LpMFG to VWFA but not the opposite direction indexed reading performance in children. In addition, the RSFC strength of VWFA-LpMFG between children and adults was comparable. Although the locations of LpMFG in two groups were different, they belonged to two separate clusters obtained from nine fMRI or transcranial magnetic stimulation (TMS) studies on the neural substrates for writing (see Supplementary Figure S2 and Supplementary Table S2). The LpMFG in children is also identified as Exner’s area (Roux et al., 2009). The Exner’s area is widely believed to be involved in writing (Exner, 1881; Roux et al., 2009, 2010; Planton et al., 2013). Roux et al. (2009) termed this region as the graphemic/motor frontal area that means this region serves as a transcoding interface between orthographic output representations and the “allographic” programming of the graphomotor movements by which handwriting can be well pre-organized. A very recent TMS study found that recognition of both printed and handwritten pseudowords but not real words was significantly affected when the activity of the Exner’s area was disrupted, indicating its causal role in reading (Pattamadilok et al., 2016).

Chinese reading is quite different from reading an alphabetic language in at least two aspects. First, there are many homophones in Chinese, leading to correspondences between one pronunciation and multiple characters with different meanings (see Li and Yip, 1998 for discussion). This means that phonological clues alone cannot be reliable sources for a lexical entry. Second, Chinese characters have complex inner structures and two-dimensional layout, i.e., a character is commonly integrated with two or three graphic components that are composed of several strokes with very different shapes, which increases the difficulty of visual analysis of characters. Therefore, high-quality orthographic representations for proficient reading require more experience with visual analysis during the reading of Chinese words. In addition to reading experience, refined visual-orthographic representations can also be partly consolidated through extensive writing.

In China, children spend much time on writing characters in school and at home. In other words, children learn how to read partly through learning how to write. Writing, as a traditional approach to Chinese literacy, can enhance awareness of spatial relations between different strokes and between different radicals in a character, strengthen the memory of stroke sequence, and is beneficial to establish the connections between graphemes and the corresponding motor components. Visual-orthographic analysis of characters and the role of writing in learning to read character might be more important in Chinese reading. A stable link between these two parts facilitates visual word processing. A hypothesis that reading depends on writing in Chinese has been proposed (Tan et al., 2005). A previous large-scale behavioral study of Chinese reading in three different sites of mainland China has also provided strong and clear evidence that the overuse of Pinyin system (an alphabetic system) affects reading development (Tan et al., 2013). In our study we found RSFC and RS-GCA patterns that are consistent with these analyses. Our findings that the RSFC between LpMFG and VWFA in both groups provide robust evidence that LpMFG plays an important role in Chinese reading. The finding that the causal influence from LpMFG to VWFA positively predicted reading performance in the child group but not the adult group indicates that the immature Chinese reading needs more modulations from the motoric activities in writing to visuo-orthographic analysis. Thus, improvement of Chinese reading proficiency in childhood needs more handwriting experience to familiarize with the inner structure of characters and to refine the visuo-orthographic analytic skills.

Existing evidence has also showed that writing areas are much involved in single letter recognition in alphabetic writing systems (e.g., Longcamp et al., 2003, 2005). However, few study has found significant neural activity within Exner’s area at word level in alphabetic reading so far. In a masked priming experiment, Nakamura et al. (2012) used cursive-style words to prime dynamically-presented cursive or static words and found that activation of Exner’s area in French reading condition was not significantly different from that in Chinese reading condition. This result seems to support the universal role of writing areas in both alphabetic and logographic reading. However, using cursive-style words as primes in the study could enhance the activity within Exner’s area in French condition because cursive-style words resemble handwritten words, which could erase the differences between Chinese and French. Considering the logographic nature of Chinese writing system, it is very likely that the writing areas are more important in Chinese reading than in alphabetic reading.

Regarding our results and relevant discussion above, we can summarize as follows. The coupling between spoken language areas and VWFA increases with age, which is common across all languages including alphabetic and logographic languages (Dehaene and Cohen, 2011; Vogel et al., 2012). In skilled readers the association between reading ability and the coupling of phonological areas with VWFA differs between alphabetic languages and logographic Chinese (see Koyama et al., 2011). Specifically, Chinese reading emphasizes complex visual analysis of characters, while English reading relies more on grapheme-phoneme conversions. In skilled Chinese reading, the activation of LSMG for grapheme-phoneme conversions is reduced and the suppression of this area’s connection with VWFA facilitates reading performance. Due to complex inner structures of Chinese characters, the high degree of homophones, and the traditional approach of learning to read by learning how to write, skilled Chinese reading involves more interactions with writing (Cao and Perfetti, 2016). On the basis of the current findings, it is likely that in skilled Chinese reading, the neurocognitive process would involve the processing of visuo-orthographic information in the VWFA, as in alphabetic languages, but this is soon passed to the LpMFG for transforming orthographic representations into motoric representation due to writing, and then to LIFG for overt or covert reading. In this view, the direct information flow from VWFA and LIFG/LSMG probably impedes reading. The suppressed activation of LSMG and the suppressed connection of LSMG with VWFA may jointly facilitate Chinese reading in developed readers. Future studies need to further examine directly the cross-language differences in the reading brain and the relationship between developmental changes of reading network and reading ability.

The current resting-state fMRI study of reading has several limitations. First, the sample size of each group was relatively small, a larger sample would increase generalization of the findings in our study. Second, comparisons between multiple age groups (e.g., child groups with 2–3 years reading experience) would reveal more details of the relationships between intrinsic couplings of VWFA-language network and reading behavior. Third, IQ scores of the adults were not measured. Because the adults were recruited from two major universities, it was possible that we compared average IQ children with above IQ adults. So it would be better to compare different groups with well-matched IQ scores in future. It would also be worthwhile to explore the potential roles of verbal and performance IQ in the developmental changes of reading circuits in future studies. Finally, direct comparison between Chinese and alphabetic reading systems at the neural level could provide more direct evidence on the differences in the reading brain.

## Author Contributions

YL, LZ and HS designed the research; YL and ZX performed the research; YL, JY and LZ analyzed the data; YL, LZ, HS and PL wrote the article.

## Conflict of Interest Statement

The authors declare that the research was conducted in the absence of any commercial or financial relationships that could be construed as a potential conflict of interest.

## Footnotes

^1^http://www.fil.ion.ucl.ac.uk/spm
