# Supplementary material for: The Relationship between Intrinsic Couplings of the Visual Word Form Area with Spoken Language Network and Reading Ability in Children and Adults
Source: Front Hum Neurosci. 2017 Jun 23;11:327. doi: 10.3389/fnhum.2017.00327 (PMC5481365; doi:10.3389/fnhum.2017.00327)
Supplement: Supplementary file 1 [file Table_1.docx]

Table S1. RSFC strength of each connection in two groups and results of two-sample t-tests for each connection.

| Connection | RSFC strength | | *t* value | *p* value |
| --- | --- | --- | --- | --- |
|  | Adults (±SD) | Children (±SD) |  |  |
| VWFA-LIFG | 0.51 (±0.20) | 0.31 (±0.22) | 3.387 | < 0.001 |
| VWFA-LSMG | 0.28 (±0.20) | 0.08 (±0.20) | 3.524 | < 0.001 |
| LIFG-LSMG | 0.30 (±0.17) | 0.14 (±0.24) | 2.705 | < 0.01 |
| VWFA-LpMFGc | 0.17 (±0.23) | 0.12 (±0.22) | 0.802 | > 0.05 |
| VWFA-LpMFGa | 0.15 (±0.22) | 0.16 (±0.19) | -0.08 | > 0.05 |
| LIFG-LpMFGc | 0.22 (±0.19) | 0.27 (±0.26) | -0.76 | > 0.05 |
| LIFG-LpMFGa | 0.19 (±0.17) | 0.19 (±0.20) | 0.044 | > 0.05 |
| LSMG-LpMFGc | -0.06 (±0.18) | 0.03 (±0.30) | -1.44 | > 0.05 |
| LSMG-LpMFGa | 0.10 (±0.20) | 0.02 (±0.19) | 1.488 | > 0.05 |
| LpMFGc-LpMFGa | 0.23 (±0.23) | 0.12 (±0.18) | 1.847 | > 0.05 |
