# Supplementary material for: The Relationship between Intrinsic Couplings of the Visual Word Form Area with Spoken Language Network and Reading Ability in Children and Adults
Source: Front Hum Neurosci. 2017 Jun 23;11:327. doi: 10.3389/fnhum.2017.00327 (PMC5481365; doi:10.3389/fnhum.2017.00327)
Supplement: Supplementary file 2 [file Table_2.docx]

Table S2. The LpMFG locations in nine fMRI or TMS studies on writing.

| Authors | Year | MNI coordinates | | |
| --- | --- | --- | --- | --- |
|  |  | x | y | z |
| Anderson et al. | 1990 | -44 | 6 | 28 |
| Longcamp et al. | 2003 | -53 | 4 | 40 |
|  |  | -28 | -1 | 65 |
| Purcell et al. | 2011 | -22 | -4 | 54 |
|  |  | -44 | 6 | 28 |
| Nakamura et al. | 2012 | -24 | -4 | 52 |
| Planton et al. | 2013 | -22 | -8 | 54 |
|  |  | -26 | 2 | 58 |
|  |  | -50 | 6 | 26 |
| Yuan & Brown | 2015 | -50 | 4 | 28 |
| Pattamadilok et al. | 2016 | -32 | 2 | 52 |
| Cao et al. | 2016 | -48 | 2 | 30 |
|  |  | -26 | -8 | 56 |
|  |  | -48 | 4 | 32 |
|  |  | -50 | 2 | 28 |
| Lagarrigue et al. | 2017 | -24 | -9 | 57 |

Anderson, S. W., Damasio, A. R., & Damasio, H. (1990). Troubled letters but not numbers: Domain specific cognitive impairments following focal damage in frontal cortex. *Brain* 113, 749-766. doi: 10.1093/brain/113.3.749

Cao, F., and Perfetti, C. A. (2016). Neural signatures of the reading-writing connection: greater involvement of writing in Chinese reading than English reading. *PLoS ONE* 11: e0168414. doi: 10.1371/journal.pone.0168414

Lagarrigue, A., Longcamp, M., Anton, J. L., Nazarian, B., Prevot, L., Velay, J. L., et al. (2017). Activation of writing-specific brain regions when reading Chinese as a second language. Effects of training modality and transfer to novel characters. *Neuropsychologia* 97, 83-97. doi: 10.1016/j.neuropsychologia.2017.01.026

Longcamp, M., Anton, J. L., Roth, M., & Velay, J. L. (2003). Visual presentation of single letters activates a premotor area involved in writing. *Neuroimage* 19, 1492-1500. doi: 10.1016/S1053-8119(03)00088-0

Nakamura, K., Kuo, W.J., Pegado, F., Cohen, L., Tzeng, O.J., and Dehaene, S. (2012). Universal brain systems for recognizing word shapes and handwriting gestures during reading. *Proc. Natl. Acad. Sci. U.S.A.* 109, 20762-20767. doi: 10.1073/pnas.1217749109

Pattamadilok, C., Ponz, A., Planton, S., & Bonnard, M. (2016). Contribution of writing to reading: Dissociation between cognitive and motor process in the left dorsal premotor cortex. *Hum. Brain. Mapp.* 37, 1531-1543. doi: 10.1002/hbm.23118

Planton, S., Jucla, M., Roux, F. E., & Démonet, J. F. (2013). The “handwriting brain”: a meta-analysis of neuroimaging studies of motor versus orthographic processes. *Cortex* 49, 2772-2787. doi: 10.1016/j.cortex.2013.05.011

Purcell JJ, Turkeltaub PE, Eden GF and Rapp B (2011) Examining the central and peripheral processes of written word production through meta-analysis. *Front. Psychol.* 2:239. doi: 10.3389/fpsyg.2011.00239

Yuan, Y., & Brown, S. (2015). Drawing and writing: An ALE meta-analysis of sensorimotor activations. *Brain Cogn.* 98, 15-26. doi: 10.1016/j.bandc.2015.05.004
