# Supplementary material for: The Relationship between Intrinsic Couplings of the Visual Word Form Area with Spoken Language Network and Reading Ability in Children and Adults
Source: Front Hum Neurosci. 2017 Jun 23;11:327. doi: 10.3389/fnhum.2017.00327 (PMC5481365; doi:10.3389/fnhum.2017.00327)
Supplement: Supplementary file 3 [file Presentation_1.pptx]

## Slide 1
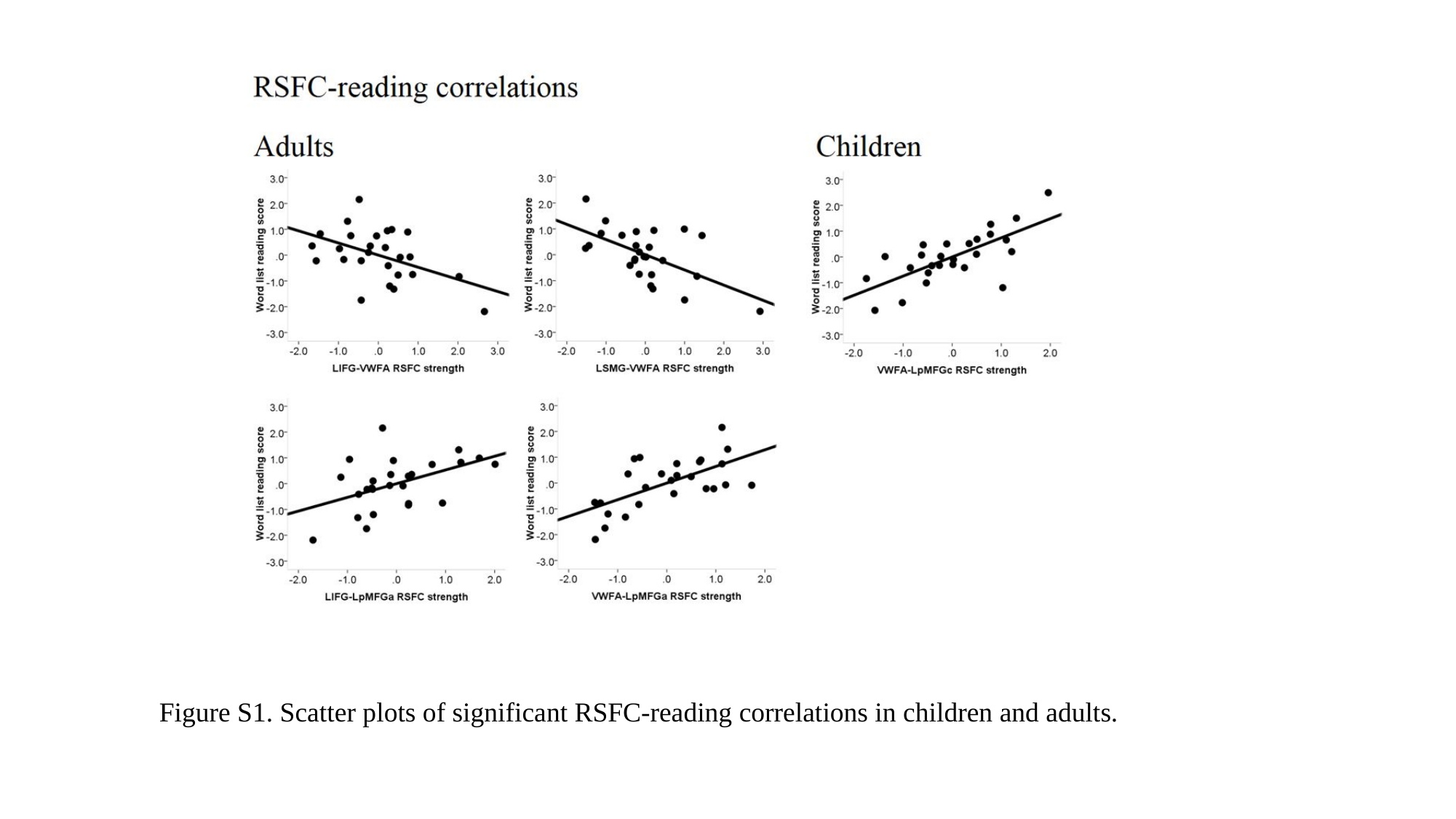

Figure S1. Scatter plots of significant RSFC-reading correlations in children and adults.

## Slide 2
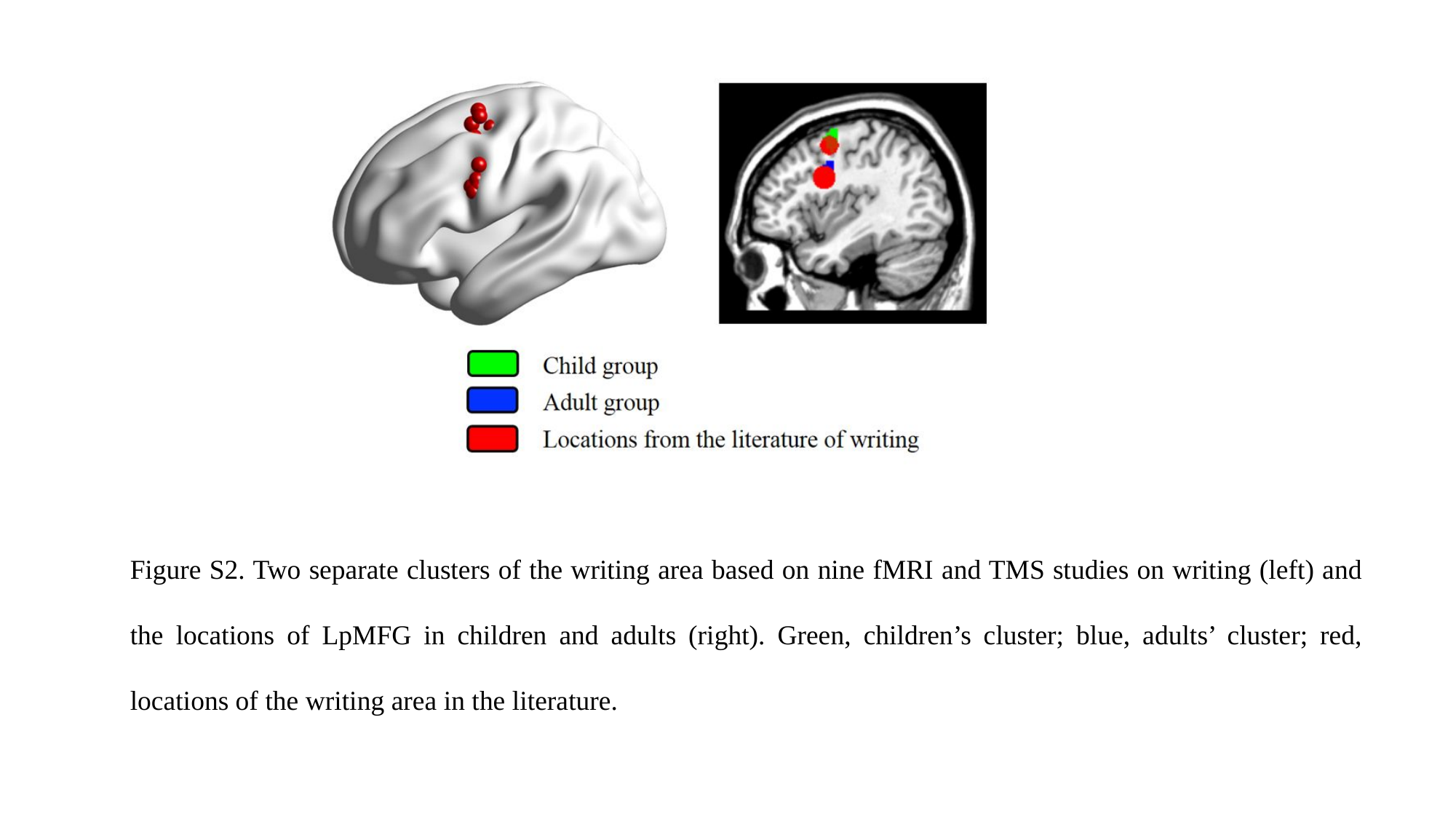

Figure S2. Two separate clusters of the writing area based on nine fMRI and TMS studies on writing (left) and the locations of LpMFG in children and adults (right). Green, children’s cluster; blue, adults’ cluster; red, locations of the writing area in the literature.

## Slide 3
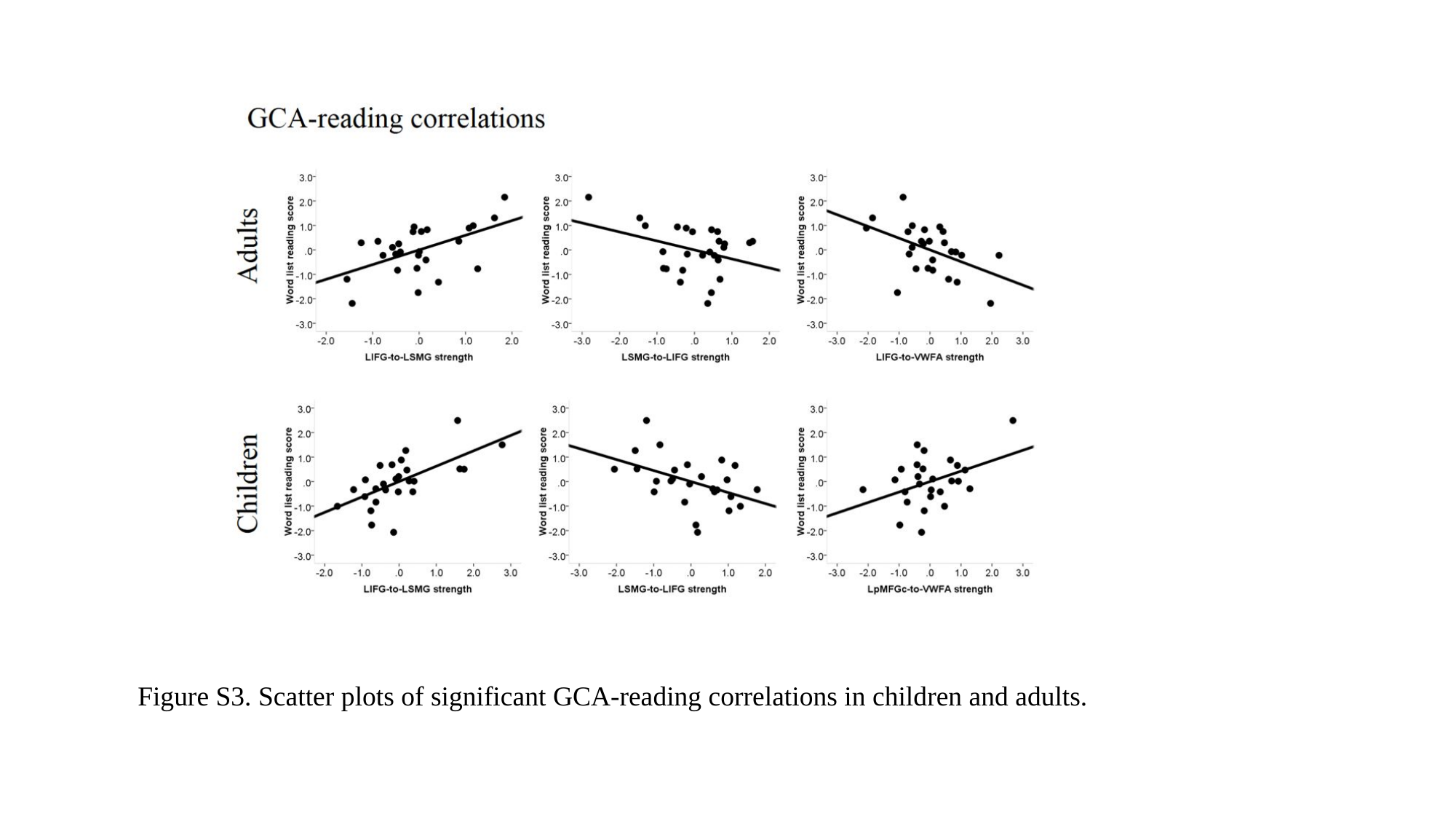

Figure S3. Scatter plots of significant GCA-reading correlations in children and adults.
